# Color diversity judgments in peripheral vision: Evidence against “cost-free” representations

**DOI:** 10.1371/journal.pone.0279686

**Published:** 2022-12-30

**Authors:** Brylee Hawkins, Dee Evans, Anya Preston, Kendra Westmoreland, Callie E. Mims, Kiara Lolo, Nicholas Rosario, Brian Odegaard

**Affiliations:** 1 Department of Psychology, University of Florida, Gainesville, Florida, United States of America; 2 Department of Psychology, University of South Alabama, Mobile, Alabama, United States of America; University of Canberra, AUSTRALIA

## Abstract

Is visual perception “rich” or “sparse?” One finding supporting the “rich” hypothesis shows that a specific visual summary representation, color diversity, is represented “cost-free” outside focally-attended regions in dual-task paradigms [1]. Here, we investigated whether this “cost-free” phenomenon for color diversity perception extends to peripheral vision. After replicating previous findings and verifying that color diversity is represented “cost-free” in central vision, we performed two experiments: in our first experiment, we extended the paradigm to peripheral vision and found that in minimally-attended regions of space, color diversity perception was impaired. In a second and final experiment, we added confidence judgments to our task, and found that participants maintained high levels of metacognitive awareness of impaired performance in minimally-attended visual areas in the periphery. These findings provide evidence that color perception may be partially attention-dependent in peripheral vision, and challenge previous views on both sides of the rich vs. sparse debate.

## Introduction

How much of the unattended visual periphery do we actually perceive? Evidence can be found to support the idea that peripheral visual perception is either better or worse than we might first suppose. As noted previously [2], studies of change blindness and inattentional blindness provide evidence that perception is sparse and limited to the few items to which we attend [3,4]. Conversely, studies of iconic memory indicate perception may be much richer than we suppose, as subjects can recall a surprisingly large number of items from briefly-presented images [5,6]. While the scientific debate surrounding this question continues, one intriguing proposal to bridge this gap has posited that peripheral vision may be characterized by the representation of summary statistics [7,8]. Far from representing details outside the fovea with a high degree of fidelity, it is thought that peripheral vision encodes a lossy compression of content which preserves gist-level information at the expense of fine-grained details.

An example of this principle can be found in color perception. It is well-established that color perception is worse in peripheral vision compared to central vision, as the number of cones outside the fovea decreases with eccentricity [9]. Despite this deficit, demonstrations reveal that if stimuli are enlarged to compensate for decreasing acuity, rudimentary color perception extends to surprisingly eccentric positions in the visual field [10]. Moreover, recent results show that observers can make judgments about a particular summary statistic, “color diversity,” outside of an attended location [1]. In a series of experiments, participants viewed a briefly-presented 4x6 rectangular grid of colored letters and were asked to recall a specific letter from a “pre-cued” row and rate the level of color diversity for different rows in the grid. Data showed that the task of reporting color diversity for “non-cued” rows did not impact people’s ability to accurately recall specific letters from cued rows, indicating color diversity may be processed “cost-free” (i.e., without a reduction in task performance) outside of attended areas in dual-task settings [1]. Building on this finding, we ask a simple question: does the phenomenon of representing color diversity “cost-free” extend to peripheral vision?

To date, results from previous studies exploring color diversity perception [1,11] have been limited in their ability to speak to peripheral vision for two reasons. First, subjects completed these tasks on computer monitors which test perception near the central visual field. Second, the location of the secondary task regarding color diversity judgments is near the location of attended information. Previous research indicates that the “useful field of view” (i.e., the area around the fixation point effectively processed in a single fixation [12]) is quite small [13,14]. Therefore, it is an open question whether findings about “cost-free” representations of color extend to paradigms where perceptual judgments are evaluated in *peripheral vision*, *further away* from the location of the primary letter recall task.

To pursue this question, we completed a series of experiments. First, we replicated the original finding from [1] on a standard computer monitor (Supplemental Experiment 1 in S1 File). Next, using the same letters, colors, and timing parameters from this task, we modified the stimulus presentation method, displaying a circular letter array on a large projector screen in the peripheral visual field (Experiment 1). Finally, we conducted an experiment with the same task but a different subjective question at the end of each trial, where we asked participants to judge their confidence in their color diversity judgments in peripheral vision (Experiment 2). Confidence ratings often correlate with reports of subjective awareness [15–17], and they have extensive utility in studies of consciousness [18], motivating our use of these measures here. To anticipate our results, in Experiment 1, we found that color diversity perception was not “cost-free” but did remain above chance-level performance in the visual periphery. In Experiment 2, observers demonstrated surprisingly high metacognitive efficiency (defined as the ability of confidence judgments to distinguish between correct and incorrect responses) in both cued and uncued regions of space. We discuss the implications of these findings for current visual theories of conscious perception in our Discussion.

## Experiment 1

Experiment 1 used the same timing parameters, stimuli, and task from [1] in an effort to determine whether color diversity (defined dichotomously as either “low” or “high”) is represented “cost-free” (i.e., without a reduction in performance in unattended areas) in peripheral regions of the visual field. Our experiment differed from [1] in two primary ways: first, instead of using a square grid of colored letters at the center of a computer monitor, we presented our stimuli on a large projection screen to extend the visual display to eccentric locations. Second, we added “catch trials” at fixation to ensure that participants were required to look at the center of the screen, to avoid making saccades to the location of the area where the attention cue appeared.

### Participants

Twenty-five undergraduate students at the University of Florida volunteered to participate in order to earn course credit. Three participants did not complete the experiment and one participant accidentally restarted the task, yielding a final sample size of twenty-one participants (16 females, 5 males, mean age = 18.57, SD = 0.93). We had previously piloted this study in 30 subjects and conducted a power analysis based on this pilot; with an effect size of 0.85, an alpha value of 0.05, and a two-tailed test, we determined that we would need 21 subjects in Experiment 1, and we fulfilled this goal. All participants gave written informed consent, and all research for this study (UF IRB #201902333) was conducted in accordance with the Declaration of Helsinki.

### Apparatus and materials

This experiment was conducted in a large experiment room using PsychoPy3 running on an Apple MacMini. Visual stimuli were presented using an Optoma HD143Z 1080p projector (60Hz refresh rate, 1080p resolution, “Vivid” and “Bright’’ display modes) on a large ShowMaven canvas projector screen. The Projector was mounted to a Gravity Pole, which was raised to a height of 221 cm, and placed 394 cm away from the projection screen. The projection screen measured 133 cm x 226 cm, and the functional projection area from the Optoma projector measured 124.5 cm x 213 cm. Subjects were seated at a table with their heads in a chinrest approximately 149 cm from the screen, which maximized the potential display area of the visual periphery while minimizing any interference from their shadow on the projected image. Ambient lighting was provided by a push-activated light in the room.

The letters used in this experiment were 9 consonants (R,T,F,N,B,P,L,M,K), with the letter for each position selected using the “choose” and “randbetween” functions in Excel. The colors of the letters were selected from the following list of 19 colors: green, olive drab, steel blue, gold, spring green, slate blue, purple, orange red, medium orchid, orchid, turquoise, violet red, blue, yellow, pink, orange, sienna, royal blue, and red. The colors for all letters in a given row were drawn from either a “low diversity” or “high diversity” setting, depending on the condition. In “low diversity” rows, the colors for the letters were sampled with replacement from a list of 6 adjacent colors on the color wheel from Bronfman et al. 2014. For “high diversity,” rows, colors were sampled with replacement from all 19 possible colors. Participants input their answers using a computer keyboard.

The circle of 24 colored letters that were shown on screen measured approximately 114cm in diameter. The diameter of the circle of letters shown on the screen measured 118cm from outside of letter to outside of letter and measured 110.4cm from inside of letter to inside of letter; each letter was 7.6cm tall. Letters were equally spaced around the circle. The subtended visual angle of our circular array totaled 43.2 degrees. The midpoint of each letter was located approximately 21 degrees away from the fixation point. Within a cued region, the distance from the top of the letter in one section to the bottom of the arc was 63.5cm.

### Procedure

**Fig 1:**
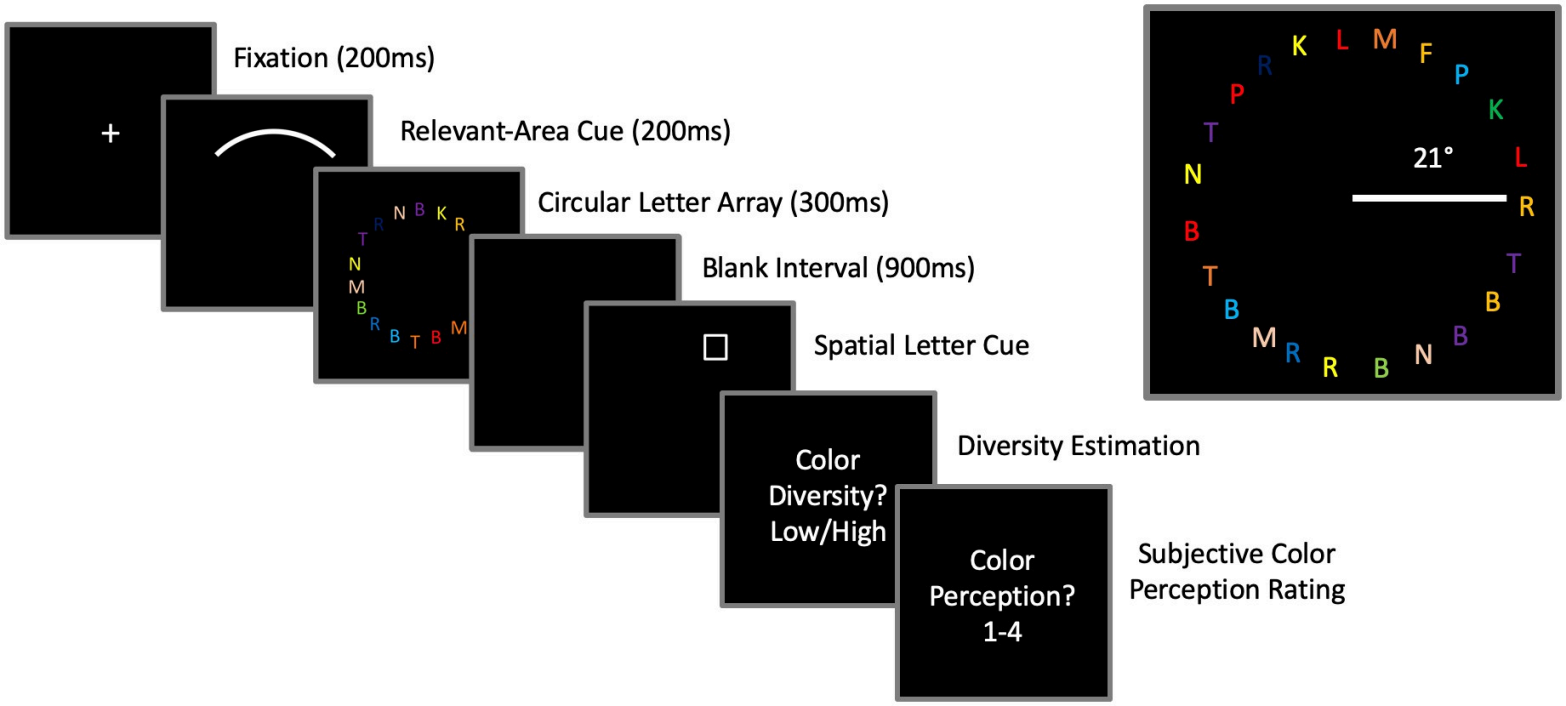
Experiment 1: Testing “cost-free” color perception in peripheral vision. On each trial, following a brief fixation cross, participants received a 200ms cue (a curved white arc) which alerted them to the region of space that they needed to attend to. Following the cue, 24 colored letters appeared in a circular pattern for 300ms in the visual periphery. As shown by the inset, these letters were presented approximately 21 degrees away from fixation on our large projection screen. Following a blank interval of 900ms, participants then answered three questions: The cued letter recall, a judgment of whether the color diversity (in the cued or uncued regions) was either low or high, and their subjective color perception for the same region as the color diversity judgment. On a small proportion of trials, the white fixation cue was accompanied by a letter placed at fixation, and subjects were immediately asked to report the letter identity. These “catch” trials were randomly interleaved throughout the experiment to ensure that subjects were incentivized to always fixate on the cross.

Our procedure followed the same timing & protocol as [1] for both the practice block and real experiment, with a few key differences (see Fig 1). First, the cue that was used in this task consisted of a curved white arc rather than an empty white rectangle. This cue designated the quarter-region of the circle that subjects needed to attend to. Each quarter-region cue (along with the corresponding group of 6 letters) was centered at the 0-degree, 90-degree, 180-degree, and 270-degree position around the circle. Second, the letters that were presented were in the form of a circle, which was presented outside foveal and parafoveal regions. The spatial letter cue for the cued recall task always corresponded to one of the six positions in the cued region of space, and the two questions asked in this task (color diversity/subjective color perception), along with the levels of the subjective rating scale, were identical to Experiment 1.

The experiment began with a “practice” block which involved only the letter-recall task for 70 trials. On each trial, participants viewed a white fixation cross at the center of the screen on a black background for 200ms. Then, a white cue (an arc on the screen) appeared for 200ms, designating the region of the stimulus circle that participants were to pay attention to. Immediately following the cue, a circular array of 24 colored letters was shown for 300ms. (The colors for each of the letters were drawn randomly from the color list in the practice block.) Following a blank interval of 900ms, the spatial letter cue (a white box) appeared, designating the letter that participants had to report from the cued area. Following completion of the practice trials, participants began the main experiment.

For the main experiment, 288 trials were conducted in the same manner as the practice block, but in addition to answering the letter-recall question, participants were required to answer two additional questions at the end of each trial (see Fig 1). One question asked participants to make a judgment about whether the color diversity in specific row(s) was (were) either low or high. Following previous studies [1,11], in the first half of trials of the experiment, participants were required to judge whether the color diversity of the *cued* row was “low” or “high;” participants indicated their response by typing either “L” or “H” on the keyboard. In the second half of trials, participants were required to judge the color diversity of the 3 *uncued* rows on each trial. The final question (asked on all trials) asked about subjects’ subjective impression of the colors in the row(s) in which they had just evaluated color diversity. Participants were asked to rate their subjective impression of the individual colors on a scale from 1–4 using numbers on the computer keyboard:

1. I had no sense that any of the letters had any color at all.
2. I had a vague sense that the letters were colored in general, but I didn’t clearly perceive the individual colors of individual letters.
3. I had a clear sense that the letters were colored in general, but I didn’t clearly perceive the individual colors of individual letters.
4. I had a clear sense that the letters were colored in general, and I could also clearly perceive the individual colors of individual letters.

As in previous research [1,11], subjects were instructed that the letter recall task was the “primary task,” but that they should still try their best on the secondary tasks of color diversity judgments and subjective ratings. On average, participants took about 5 minutes to complete the practice trials and 40 minutes to complete the experimental trials. Participants were given an opportunity to take a break every 96 trials.

We note that to maintain consistency with [1,11], we did not enforce fixation throughout the trial in this new setup. However, to ensure that observers were maintaining fixation, we added sixty “catch trials,” where upon the presentation of the peripheral cue, the fixation cross was replaced with a letter. These trials immediately terminated following the presentation of the letter, and subjects were asked to report the letter’s identity, which could be any one of the nine consonants described above.

### Results

**Fig 2:**
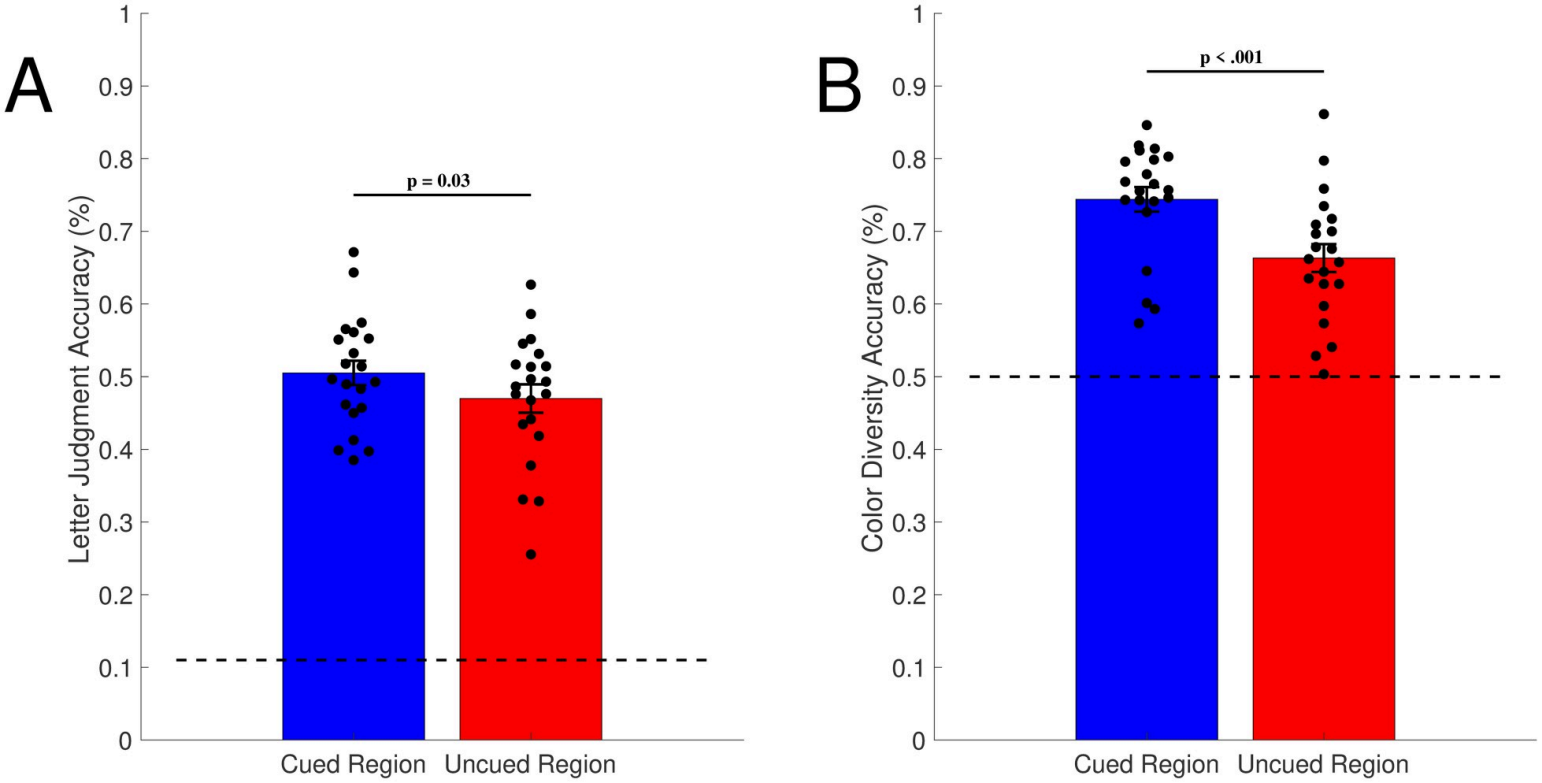
Experiment 1: Average accuracy across subjects for letter recall and color diversity judgments. The mean of all subject averages is shown, with error bars showing SEM across subjects. Dotted lines represent chance-level performance. (A) Letter Recall Task. The average accuracy across subjects for the letter recall task was significantly higher when the color diversity question focused on the cued region of the circle, compared to when this question queried the uncued region of the circle. (B) Color diversity judgments. The average accuracy for color diversity was higher for cued regions of the circle compared to uncued regions of the circle.

For each observer, we first computed average letter judgment accuracy on trials where subjects had to report the color diversity of the cued row, and letter judgment accuracy on trials where they reported color diversity of the uncued row. As shown in Fig 2A, there was a difference in letter judgment accuracy for the two conditions (50.5% vs. 47.0%, respectively), and across both conditions, letter judgment performance was considerably greater than chance (11.11%). To test whether the difference between conditions was significant, we conducted a Shapiro-Wilk test of normality, which did not suggest a deviation from normality (W = 0.97, p = .65); therefore, we conducted a paired-samples t-test, which showed that the difference was significant (t(20) = 2.4, p = 0.03, Cohen’s *d* = 0.53).

As shown in Fig 2B, participants were also able to perform the color diversity judgments effectively for both cued rows and uncued rows (74.4% vs. 66.3%, respectively; chance level 50%). However, several interesting findings emerged. First, to test whether this difference in color judgment performance between conditions was significant, we conducted a Shapiro-Wilk test of normality, which suggested a deviation from normality (W = 0.89, p = .02); considering the non-normality of our data, we then conducted a Wilcoxon signed-rank test, which indicated that the accuracy of the color diversity judgment was higher for cued regions than uncued regions (W = 208.00, p < .001). This result provides evidence that observers may not represent color diversity in an attention-independent, “cost-free” manner throughout the visual surround (Bronfman et al. 2014). It is interesting to note that even though color diversity was impaired in the uncued regions, performance remained above chance (66.3% compared to 50%). This indicates that even in minimally-attended regions of peripheral visual space, participants may still preserve *some* color diversity perception, even if it isn’t “cost-free.”

**Fig 3:**
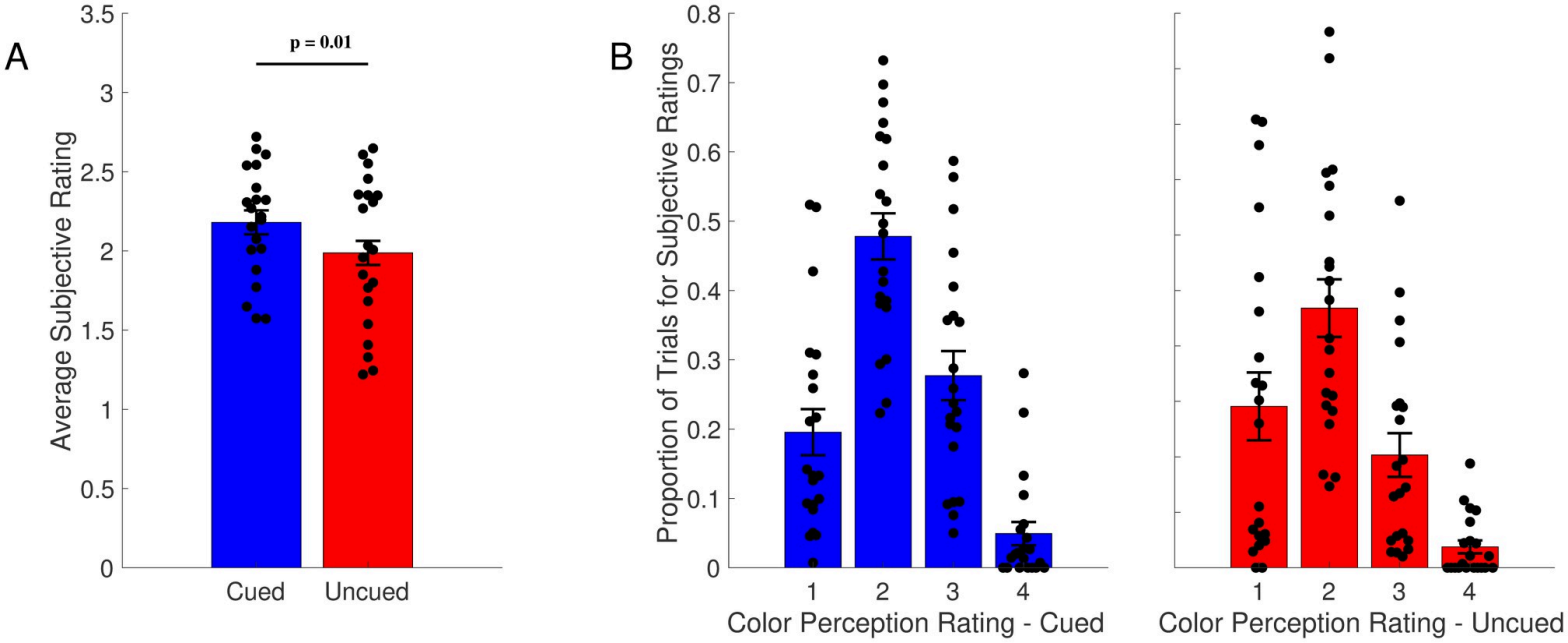
Subjective ratings for color diversity judgments in Experiment 1. (A) Average subjective rating for color diversity judgments for the cued and uncued rows. Subjective ratings for uncued rows were significantly lower than cued rows. (B) Proportion of trials for each subjective rating type. The main difference between ratings for the cued and uncued trials were differences in the frequencies of the “1” and “3” responses. Error bars show SEM across subjects.

Subjective measures from our secondary questions revealed that observers reported seeing uncued rows less vividly than cued rows. As shown in Fig 3A, subjects reported an average rating of 2.18 for cued rows, and 1.99 for uncued rows. A Shapiro-Wilk test of normality did not suggest a deviation from normality (W = 0.97, p = .75); thus, we conducted a paired-samples t-test, which revealed that the difference in subjective ratings between cued and uncued rows was significant (t(20) = 3.11, p = 0.01, Cohen’s *d* = 0.68). The proportion of trials with different subjective ratings for the two conditions is plotted in Fig 3B.

We also note that catch trial performance in this task was quite high (mean 96.2%; std deviation .035), with all observers above 85%, indicating fixation compliance was quite high.

## Experiment 2

Experiment 1 revealed an interesting finding: contrary to general claims by [1], color diversity does not appear to be perceived “cost-free” in the visual periphery. In Experiment 1, we used the same task, stimuli, and subjective rating scale as their original paper. In Experiment 2, we used the exact same task to probe a different subjective measure: perceptual confidence. Since perceptual confidence provides an alternative measure of metacognitive awareness, we aimed to measure the degree to which confidence tracks the accuracy of color diversity judgments outside the center of gaze.

### Participants

Our sample consisted of 26 undergraduate students at the University of Florida who volunteered to participate in this research study in order to earn course credit. Of these 26 participants, five were excluded due to either task negligence or responding with the same level of confidence on each trial. Thus, we analyzed data from 21 participants for this study. This sample of participants consisted of 8 males, 12 females, and one person abstaining from reporting biological sex. The mean age was 19.47 years, SD = 1.93. All participants gave written informed consent, and all protocols (UF IRB #201902333) were conducted in accordance with the Declaration of Helsinki.

### Apparatus, materials, and procedure

The experimental setup, design, and overall procedure was identical to Experiment 1. However, the one difference in this experiment was that at the end of each trial, instead of asking for a subjective rating of the perception of individual colors using a scale from 1–4, we asked participants to judge their confidence in their color diversity judgment on a scale from 1–4. “1” designated “not at all confident, and “4” designated “extremely confident.” Critically, this experiment also contained the same catch trials from Experiment 1, to ensure that participants were incentivized to maintain fixation at the center of the screen.

### Results

In Experiment 2, we replicated our primary findings from Experiment 1. For the letter recall task, performance was higher for the cued regions of the circle compared to the uncued regions of the circle (50.8% vs. 45.1%). A Shapiro-Wilk test suggested a deviation from normality (W = 0.90, p = .03); therefore, we conducted a Wilcoxon signed-rank test, which showed that the difference was significant (W = 202.00, p = .002). For the color diversity question, judgments for the cued regions of the circle were significantly more accurate compared to judgments for the uncued regions of the circle (79.0% vs. 67.3%). A Shapiro-Wilk test did not suggest a deviation from normality (W = 0.95, p = .36), so we conducted a paired-samples t-test, which revealed this difference was significant (t(20) = 6.85, p < .001, Cohen’s *d* = 1.50). Next, we analyzed confidence ratings for the color diversity judgments. As expected, on average, confidence ratings were higher for color diversity judgments in the cued region (mean = 2.94; SD = 0.31), compared to the uncued region (mean = 2.58; SD = 0.48). A Shapiro-Wilk test did not suggest a deviation from normality (W = 0.96, p = .47), so we conducted a paired-samples t-test, which revealed this difference was significant (t(20) = 4.62, p < .001, Cohen’s *d* = 1.01).

**Fig 4:**
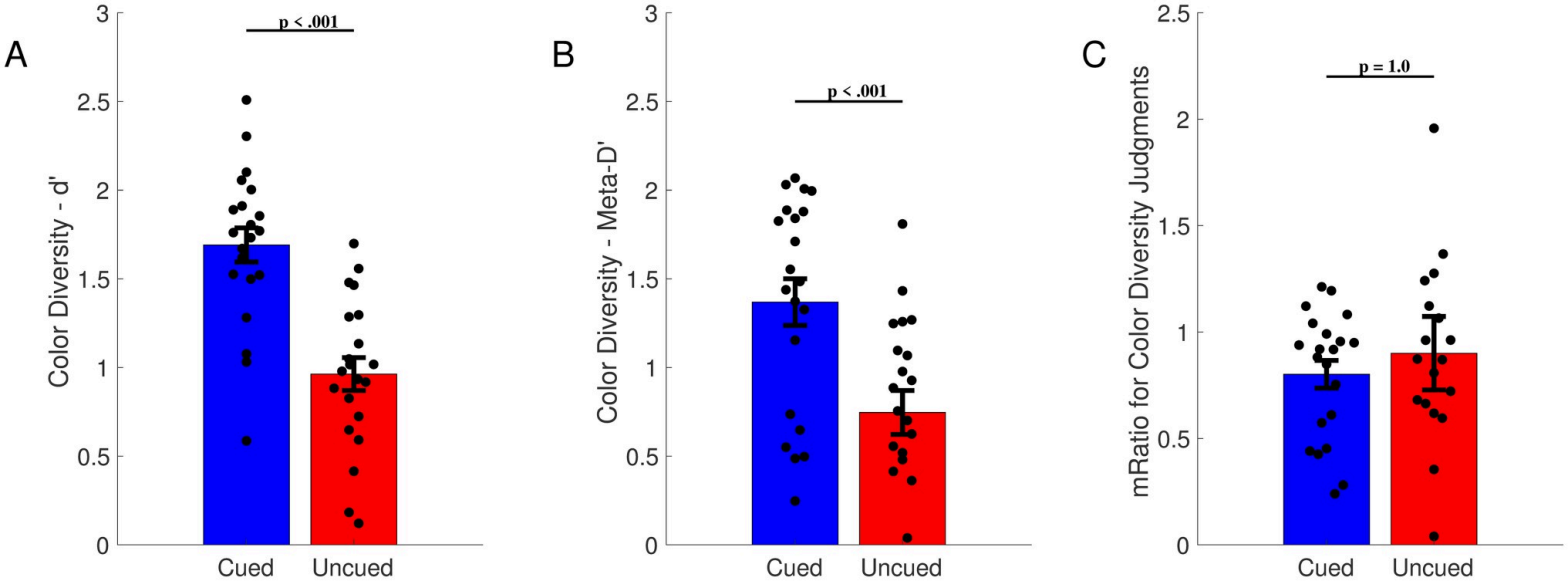
Measures of metacognition for color diversity judgments in Experiment 2. The mean of all subject averages is shown, with error bars showing SEM across subjects. (A) *d’* for color diversity judgments. (B) Meta-d’ for color diversity judgments. This measure was significantly higher for cued compared to uncued regions of the circle. (C) The M-ratio. This measure of metacognitive efficiency was nearly identical for cued and uncued regions of the circle. Note that the high p-value is from Wilcoxon test.

Next, we computed the “M-ratio” for the color diversity judgments [19]. The purpose of this measure was to produce a normalized measure of metacognitive efficiency. We began this process by computing d’ for color diversity judgments in cued and uncued regions of the circle (Cued Avg: 1.69, Uncued Avg: 0.96; t(20) = 6.44, p < .001, Cohen’s *d* = 1.41; Fig 4A). Next, we computed Meta-d’ for color diversity judgments in cued and uncued regions. Meta-d’ is a measure of how effectively confidence judgments track trial-by-trial accuracy (Maniscalco and Lau 2012). Overall, meta-d’ was higher in cued regions than in uncued regions (Cued Avg: 1.37, Uncued Avg: 0.75; t(20) = 4.82, p < 0.001, Cohen’s *d* = 1.05; Fig 4B). Finally, we computed the M-ratio, which involves dividing meta-d’ by d’. As can be seen in the Fig 4C, the M-ratio was similar for the cued and uncued regions of the circle (Cued Avg: 0.80, Uncued Avg: 0.90). A Shapiro Wilk Test revealed a significant deviation from normality; therefore, we computed a Wilcoxon Rank-Sum Test, which showed that the difference between cued and uncued location M-ratios was not significant (W = 116.00, p = 1.00).

**Fig 5:**
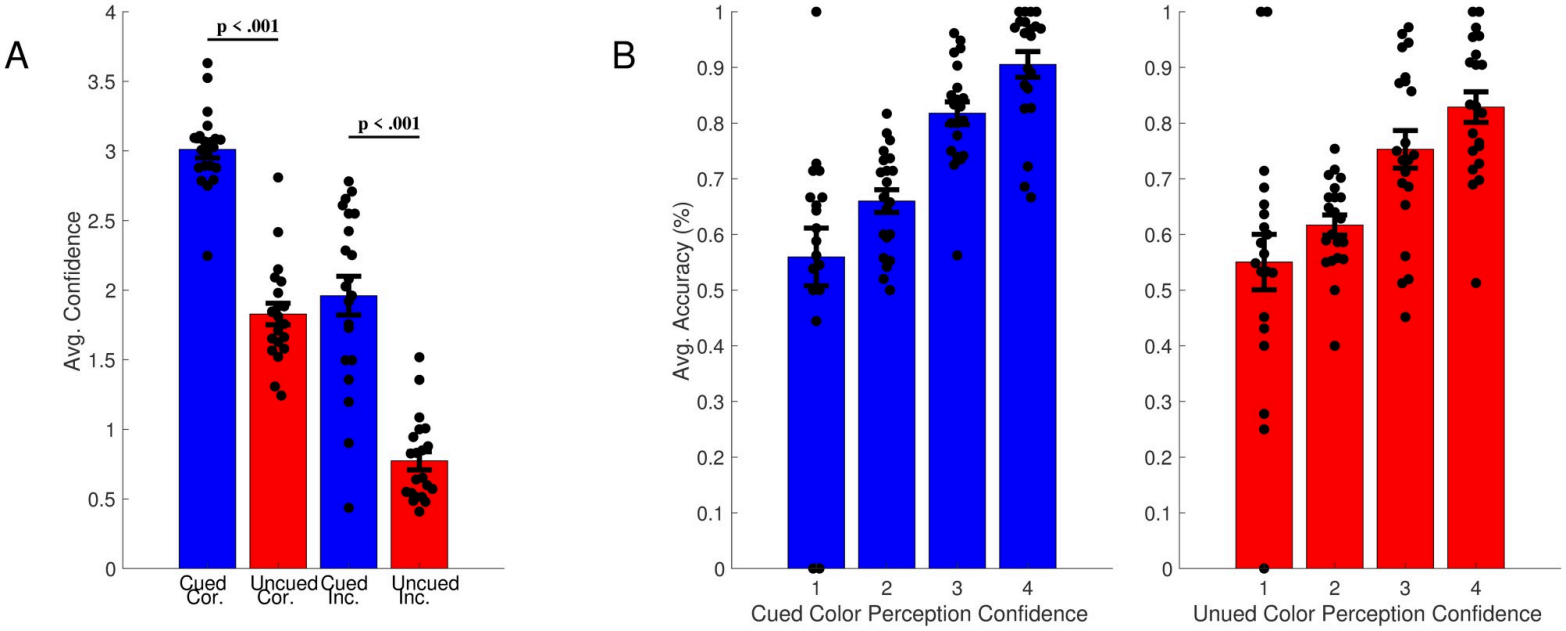
Confidence for correct and incorrect color diversity judgments in Experiment 2. (A) Average confidence for cued and uncued color diversity judgments, separated by whether the judgment is correct or incorrect. As can be seen in the figure, in general, confidence was higher for correct than incorrect judgments, and this was true for both cued and uncued regions of the circle. (B). Average accuracy across color diversity judgments with different confidence levels, for cued and uncued regions. In general, accuracy increased as confidence increased for color diversity judgments in both cued and uncued regions. Error bars show SEM across subjects.

As shown in Fig 5A, in general, participants were more confident when they were correct, and less confident when they were incorrect; this trend held for color diversity judgments in both cued and uncued regions of the circle. We also compared differences between cued and uncued trials, based on whether the participant was correct or not. For correct trials, on average, confidence was significantly higher in the cued condition compared to the uncued condition (3.01 vs. 1.83; t(20) = 7.95, p < .001, Cohen’s *d* = 1.74; Fig 5A). For incorrect trials, the average confidence for cued rows (1.96) was also higher than that of uncued rows (0.77); this difference was also significant (t(20) = 11.13, p < .001, Cohen’s *d* = 2.43; Fig 5A). In general, as confidence increased, the average accuracy for trials with a given confidence rating increased (Fig 5B).

As in Experiment 1, catch trial performance in this task was quite high (mean 94.8%; std deviation .06), indicating fixation compliance was high across participants.

## Discussion

Is our perception of the visual word inherently *rich*, but limited by processing constraints from attention and memory [20,21]? Or is our phenomenological awareness of peripheral visual content sparser than we may suppose based (at least in part) on illusory contents [22,23]? Recent data have been interpreted to support the idea of phenomenological richness [24] by demonstrating that the visual system represents information “cost-free” outside areas of focal attention near the center of gaze [1]. While contemporary debate surrounding this finding has primarily focused on whether observers actually maintain differentiated representations of the colors used in this particular task [11,25,26], here, we asked the following question: do “cost-free” representations for color diversity extend to peripheral vision? It is still claimed that observers experience and discriminate color diversity outside focal attention, but to date, no studies have probed how far discrimination ability “outside focal attention” extends. Peripheral vision outside the rod-free fovea makes up 99% of the visual field, and its deficits in color vision are often overstated [27]. The entire rich vs. sparse debate is *primarily about peripheral vision*; thus, it is critical to systematically probe eccentric vision to make progress in this debate.

To answer our question of interest about peripheral vision, we conducted three experiments: first, we replicated findings demonstrating “cost-free” color diversity representations in central vision (see Supplemental Experiment 1 in S1 File) [1,11]. Second, using the same colors, timing parameters, and general design from the first experiment, we conducted a new experiment using a circular letter array in peripheral vision (21° from fixation). Our findings show that when peripheral vision is probed, and the separation between cued and uncued regions is increased, color diversity perception comes with a cost: judgments of color diversity in uncued regions of space showed reduced performance compared to making these judgments for cued regions. Additionally, these performance deficits were accompanied by changes in subjective ratings about how effectively the individual colors were perceived. Third, using the same peripheral vision task (but incorporating confidence judgments for the subjective measure), we showed that the M-ratio, a standardized measure of metacognitive efficiency which controls for perceptual sensitivity, was remarkably consistent for both cued and uncued areas, demonstrating that observers possessed (at least partial) awareness of this metacognitive deficit in unattended areas of the visual surround.

How do these findings inform the rich vs. sparse debate? On the one hand, it seems as though perception may not be as “rich” as previously claimed [2,24]: based on our results from Experiment 1 and 2, it appears that “cost-free” representations outside of focal attention may not generalize to peripheral vision, and may not generalize to circumstances with greater spatial separation between cued and uncued regions. Thus, it seems reasonable to posit that in order to maintain high-fidelity representations of summary statistical content, attention likely plays a critical role [28]. We do note, however, that even in trials in uncued regions in Experiment 1, observers’ color diversity judgments were above chance. This indicates that peripheral vision maintains *some* capacity for color diversity judgments outside of cued regions for the primary letter-recall task, but that capacity may not be as strong as previous proponents of the “rich” view have claimed. Moving forward, we recommend that future research manipulates attention in a systematic manner using valid and invalid trials. That is, the instruction to make letter recall the “primary task” and color diversity/subjective rating judgments the “secondary task” [1,11] inherently carries ambiguity that could be removed by using standard attentional cueing paradigms [29] with fixation enforced throughout the perceptual judgment, or even one-shot invalid-attention paradigms [30–32]. We hypothesize that the performance deficits in peripheral vision we observe here would be even more pronounced as the amount of allocated attention decreases.

On the other hand, it seems as though perception may not be as “sparse” as previously claimed, either. As mentioned previously, color diversity judgments in uncued regions were above chance, and as shown in Experiment 2, observers maintained a remarkable degree of metacognitive awareness of this performance deficit. One recent theoretical account has posited that perception of the visual periphery might be “inflated” as observers overestimate how well they see things in the visual surround [33,34]. From the results in Experiment 2 in the present manuscript, it appears observers possess remarkably stable metacognitive knowledge for color diversity across the visual field, posing a distinct challenge for whether the phenomenon of “inflation” extends to more complex perceptual features like color diversity. Overall, the ability for metacognitive judgments to distinguish between correct and incorrect responses for color diversity judgments was quite consistent across cued and uncued regions of space.

Finally, we think these findings also can inform recent ideas about whether attention alters appearance [35–37]. In Experiment 1, we demonstrated not only performance differences between judgments in cued and uncued regions of space, but also differences in the subjective ratings for how clearly observers perceived the individual colors. We think these findings contribute to a growing consensus that whatever is represented in the visual periphery, it is clearly influenced by attentional processes, and unattended regions are experienced with less fine-grained detail.

Moving forward, we think that all studies of peripheral vision can be improved by incorporating two elements: virtual reality and eye tracking. Recent efforts to study peripheral vision are marked by either lack of fixation controls [38] or justification of a lack of eye tracking by selecting brief stimulus durations less than 300ms, which appear to parallel how long fixations last in natural scenes [39]. In this investigation, we ensured fixation at a central location by incorporating catch trials which required observers to identify brief, centrally-presented letters. We think to further probe the richness of the visual periphery, technology which incorporates both a wider field of view (as in virtual reality) and real-time monitoring of fixation location will be critical to more precisely probe what is perceived in the visual surround, and how eye movements influence perception of items in unattended areas. Commercial devices such as the HTC Vive Pro offer one promising option to overcome these limitations in previous studies, and we think their use will be paramount moving forward to characterize perception outside the center of the visual field. Finally, we note that future studies should aim to disentangle effects due to cuing and effects due to peripheral vision. In the present work, all stimuli are presented in peripheral vision, and thus it is difficult to determine which effects are solely driven by cues, and which effects may be due to processing that is unique to peripheral vision. Additional behavioral work is necessary to systematically tease apart the specific effects of these two factors.

## Supporting information

S1 File (DOCX)
