# Supplementary material for: Color diversity judgments in peripheral vision: Evidence against “cost-free” representations
Source: PLoS One. 2022 Dec 30;17(12):e0279686. doi: 10.1371/journal.pone.0279686 (PMC9803108; doi:10.1371/journal.pone.0279686)
Supplement: S1 File — (DOCX) [file pone.0279686.s001.docx]

### **Supplemental Experiment 1**

Our very first experiment investigated color diversity perception by replicating [Bronfman et al. (2014)](https://paperpile.com/c/iIY8Nx/VP6Q), while also probing participants’ perceptions of the individual colors in the display by adding a question about the subjective perception of individual colors [(Ward, Bear, and Scholl 2016)](https://paperpile.com/c/iIY8Nx/OpVY). This was done to ensure if any differences were shown in our peripheral vision experiment, we could rule out any influence of timing and stimulus-related parameters.

**Materials & Methods**

*Participants*

Thirty undergraduate students at the University of Florida (27 women, 3 men, mean age = 18.2, SD = 0.52) volunteered to participate in order to earn course credit.

*Apparatus & Materials*

Stimuli were presented on a Gateway VX900 CRT monitor set to a refresh rate of 85 Hz and a resolution of 1280 x 1024, using PsychoPy3 running on a MacMini. Participants were seated with their head resting in a UHCO chinrest located approximately 50cm away from the monitor. On each trial, subjects were presented with a 4 x 6 grid of colored letters in Courier font in the center of the screen. The letters used in this experiment were 9 consonants (R,T,F,N,B,P,L,M,K), with the letter for each grid position selected using the “choose” and “randbetween” functions in Excel. The colors of the letters were selected from the following list of 19 colors: green, olive drab, steel blue, gold, spring green, slate blue, purple, orange red, medium orchid, orchid, turquoise, violet red, blue, yellow, pink, orange, sienna, royal blue, and red. The colors for all letters in a given row were drawn from either a “low diversity” or “high diversity” setting, depending on the condition. In “low diversity” rows, the colors for the letters were sampled with replacement from a list of 6 adjacent colors on the color wheel. For “high diversity,” rows, colors were sampled with replacement from all 19 possible colors. Participants input their answers using a computer keyboard.

*Procedure*

The experiment began with a “practice” block which involved only the letter-recall task for 70 trials. On each trial, participants viewed a white fixation cross at the center of the screen on a black background for 200ms. Then, the row cue appeared for 200ms, designating the row that participants were to pay attention to. Immediately following the cue, the 4 x 6 grid of colored letters was shown for 300ms. (The colors for each of the letters were drawn randomly from the color list in the practice block.) Following a blank interval of 900ms, the spatial letter cue (a white box) appeared, designating the letter that participants had to report. Following completion of the practice trials, participants began the main experiment.

For the main experiment, 288 trials were conducted in the same manner as the practice block, but in addition to answering the letter-recall question, participants were required to answer two additional questions at the end of each trial (see Figure S1). One question asked participants to make a judgment about whether the color diversity in specific row(s) was (were) either low or high. Following previous studies [(Bronfman et al. 2014; Ward, Bear, and Scholl 2016)](https://paperpile.com/c/iIY8Nx/VP6Q+OpVY), in the first half of trials of the experiment, participants were required to judge whether the color diversity of the *cued* row was “low” or “high;” participants indicated their response by typing either “L” or “H” on the keyboard. In the second half of trials, participants were required to judge the color diversity of the 3 *uncued* rows on each trial. The final question (asked on all trials) asked about subjects’ subjective impression of the colors in the row(s) in which they had just evaluated color diversity. Participants were asked to rate their subjective impression of the individual colors on a scale from 1-4 using numbers on the computer keyboard:

(1) I had no sense that any of the letters had any color at all.

(2) I had a vague sense that the letters were colored in general, but I didn’t clearly perceive the individual colors of individual letters.

(3) I had a clear sense that the letters were colored in general, but I didn’t clearly perceive the individual colors of individual letters.

(4) I had a clear sense that the letters were colored in general, and I could also clearly perceive the individual colors of individual letters.”

As in previous research [(Bronfman et al. 2014; Ward, Bear, and Scholl 2016)](https://paperpile.com/c/iIY8Nx/VP6Q+OpVY), subjects were instructed that the letter recall task was the “primary task,” but that they should still try their best on the secondary tasks of the color diversity judgment and subjective rating. On average, participants took about 5 minutes to complete the practice trials and 40 minutes to complete the experimental trials. Participants were given an opportunity to take a break every 96 trials.


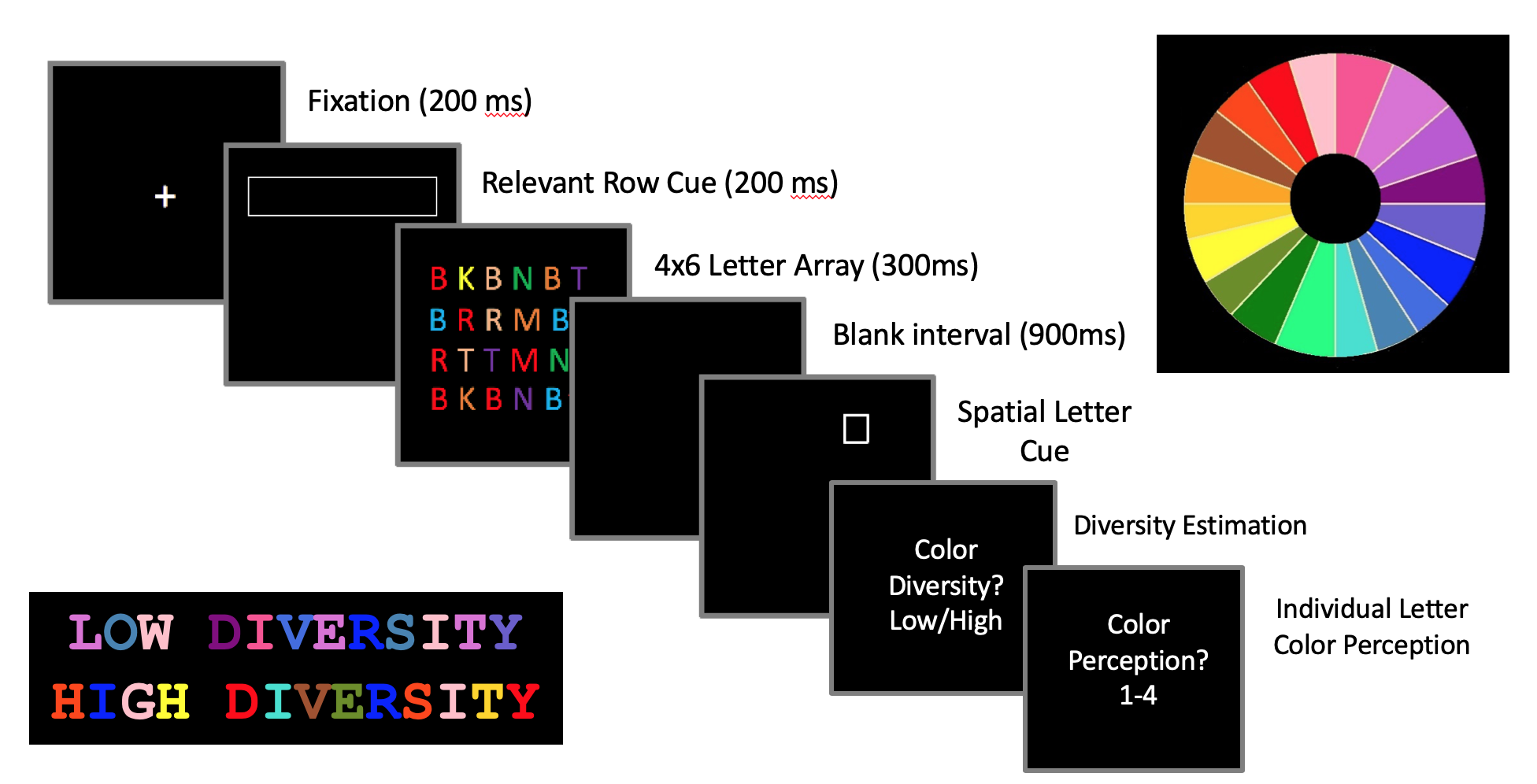


*Figure S1*. Experiment S1 Trial Structure. Each trial began with subjects fixating on a white cross, followed shortly after by a white rectangle which cued the row that needed to be remembered for the working memory task. Following presentation of the letter grid and a short blank, a white box appeared to label the specific letter that subjects needed to recall. The trial concluded with two questions: (1) a judgment of the color diversity in either the cued row (during the 1st half of trials) or uncued rows (during the 2nd half of trials), and (2) a rating of the subjective impression of the colors during the trial. The upper right inset shows the 19 possible colors of the letters that were shown; the lower left inset shows examples of the “low diversity” and “high diversity” conditions.

Finally, we note that in this task, we included the critical emphasis that subjects were to treat the letter recall task as the *primary task*, and the color diversity judgment as the *secondary task*.

**Results**

Following previous research [(Ward, Bear, and Scholl 2016)](https://paperpile.com/c/iIY8Nx/OpVY), for each observer, we first computed average letter judgment accuracy on trials where subjects had to report the color diversity of the cued row, and letter judgment accuracy on trials where they reported color diversity of the uncued row. As shown in Figure S2A, letter judgment accuracy was quite consistent between these two conditions (56.0% vs. 54.0%, respectively), and did not significantly differ between these two trial types (t(29) = 1.635, p = 0.11, Cohen’s *d* = 0.30, BF_10_ = 0.64). Performance in both conditions was also considerably greater than chance-level performance (11.11%). As shown in Figure S2B, participants were also able to perform the color diversity judgments remarkably well for both cued rows and uncued rows (70.1% vs. 70.7%, respectively), and performance did not significantly differ between these two trial types (t(29) = -0.3, p = 0.76, Cohen’s *d* = -0.06, BF_10_ = 0.2). Together, these findings replicate previous research which demonstrates how observers can evaluate color diversity outside regions of focal attention when stimuli are presented near central regions of visual space [(Bronfman et al. 2014; Ward, Bear, and Scholl 2016)](https://paperpile.com/c/iIY8Nx/VP6Q+OpVY)*.*


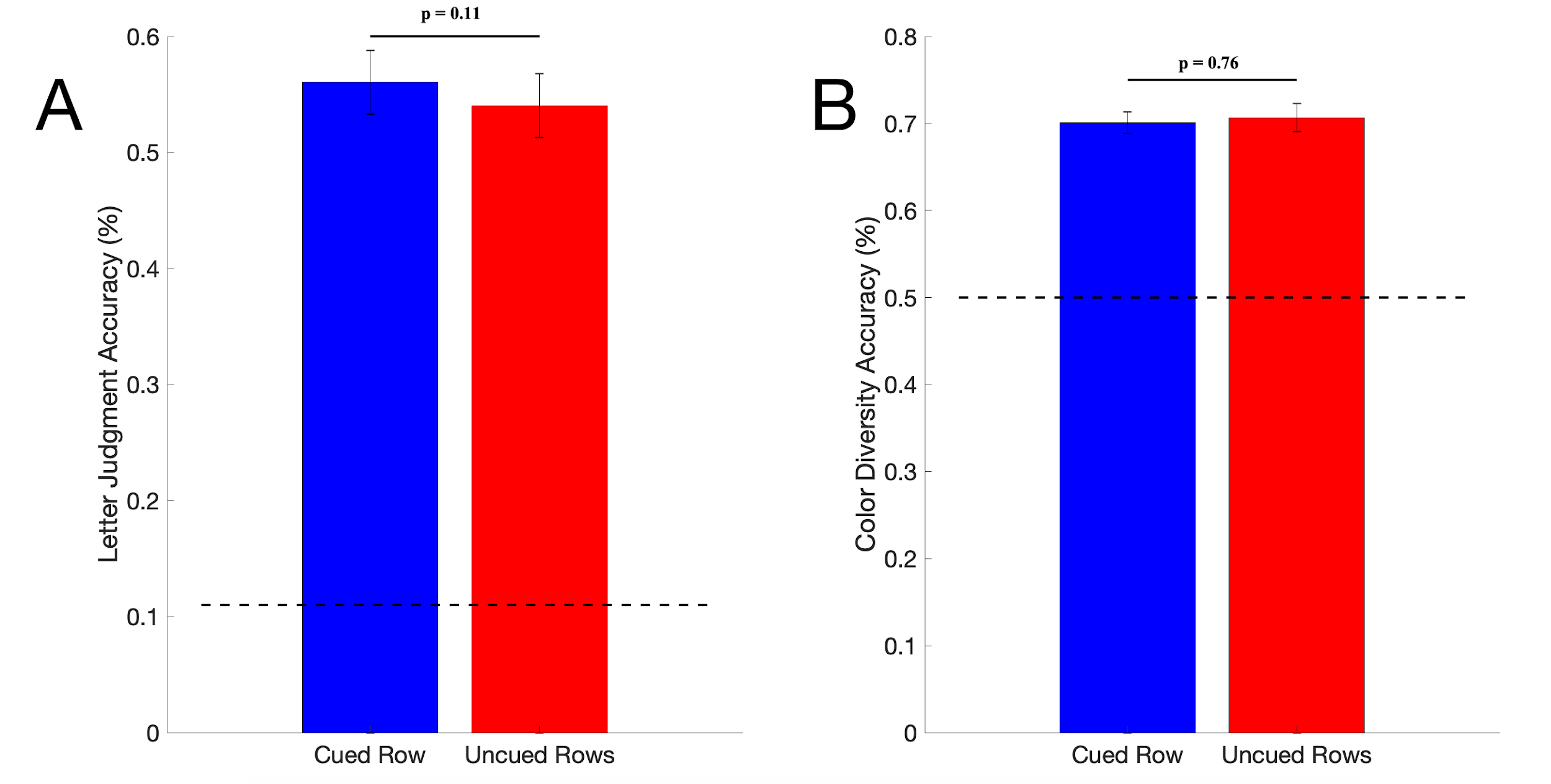


*Figure S2.* Average accuracy across subjects for letter recall and color diversity judgments in Supplemental Experiment S1. The mean of all subjects’ averages is shown, with errors bars showing SEM across subjects. (A) Letter judgment accuracy for letter recall on trials where the color diversity question queried the cued row (blue) or uncued row (red). Chance-level performance is shown by the black dotted line. (B) Color diversity accuracy. In the first half of trials, subjects reported the color diversity accuracy for the cued row, and in the second half of trials, reported color diversity accuracy for the uncued row.

Subjective measures from our secondary questions revealed that observers reported seeing uncued rows slightly less vividly than cued rows. For instance, as shown in Figure S3A, subjects reported an average rating of 2.82 for cued rows and 2.68 for uncued rows; this trend did not quite reach significance (t(29) = 1.85, p = 0.07, Cohen’s *d* = 0.34, BF_10_ = 0.88). Further inspection of the frequency of the different ratings revealed that this difference between cued and uncued rows appeared to be driven mainly by the use of the “1” and “4” ratings on the scale, rather than usage of the middle of the rating scale (Figure S3B).


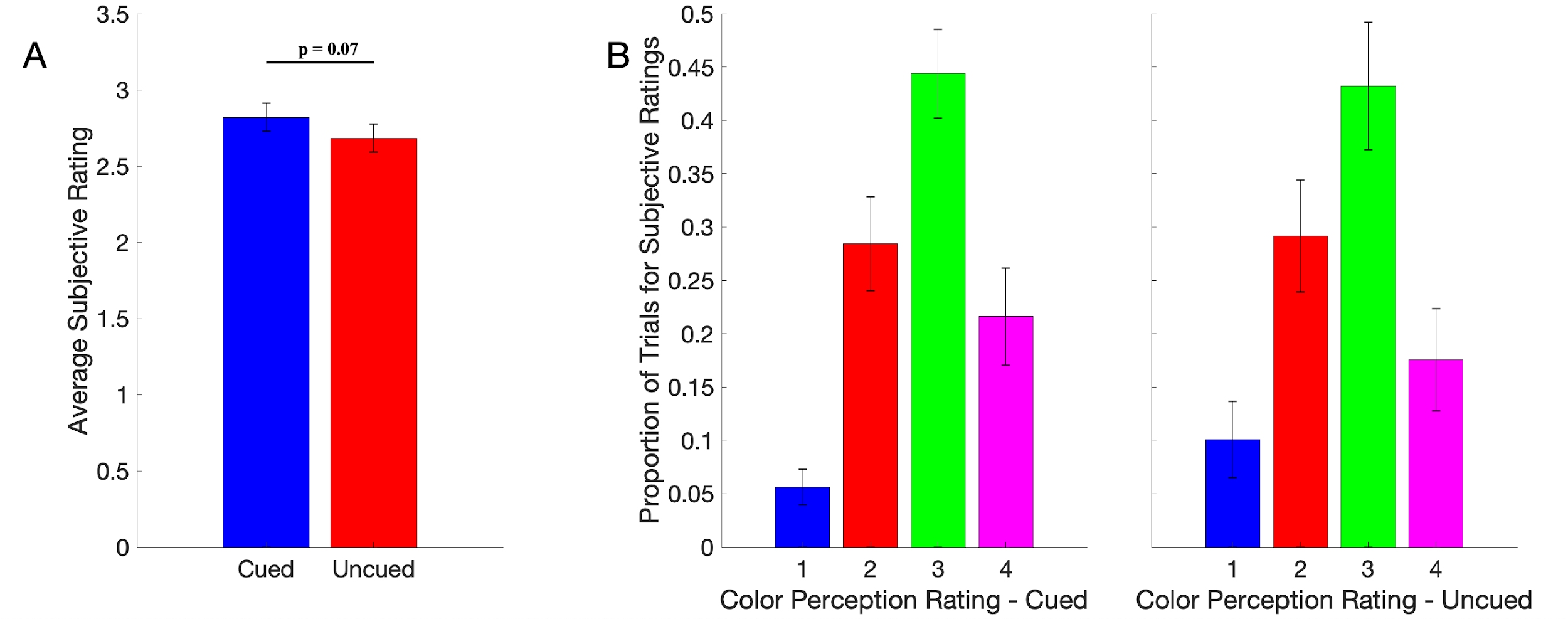


*Figure S3*. Subjective Ratings for Color Diversity Judgments in Supplemental Experiment 1. (A) Average subjective rating for color diversity judgments for the cued and uncued rows. A slight trend for reduced subjective ratings for uncued rows compared to cued rows was evident. (B) Proportion of trials for each subjective rating type. The main difference between ratings for the cued and uncued trials were small differences in the frequencies of the “1” and “4” responses.
